# Mirror, Mirror on the Wall: A Meta-Analysis on the Validity of Self-Assessed Intelligence through the Lens of the Multiverse

**DOI:** 10.3390/jintelligence12090081

**Published:** 2024-08-28

**Authors:** Sabine Patzl, Sandra Oberleiter, Jakob Pietschnig

**Affiliations:** 1 Chair of Personality Psychology and Psychological Assessment, University of Bamberg, 96047 Bamberg, Germany; sabine.patzl@uni-bamberg.de; 2 Vienna Doctoral School in Cognition, Behavior, and Neuroscience (VDS CoBeNe), University of Vienna, 1030 Vienna, Austria; sandra.oberleiter@univie.ac.at; 3 Department of Developmental and Educational Psychology, Faculty of Psychology, University of Vienna, 1010 Vienna, Austria

**Keywords:** self-assessed intelligence, meta-analysis, effect inflation, multiverse analysis, specification curve

## Abstract

Examining the relationship between self-assessed intelligence (SAI) and psychometric intelligence (IQ) is essential for understanding how people’s self-evaluations reflect their actual intelligence. Various factors, such as SAI measurement methods, participant characteristics, and testing conditions have been hypothesized to moderate the SAI–IQ link, yet the generality of this association remains unclear. Here, we provide evidence for SAI and IQ associations based on 278 effect sizes from 115 independent samples (*N* = 36,833) using a multi-level meta-analysis, revealing a moderate positive correlation (*r* = 0.30; 95% CI [0.27, 0.33]). Multiverse analyses demonstrated remarkable stability of this effect, with most summary effect specifications yielding significant positive correlations (96%), averaging *r* = 0.32. Notably, ability domain and sample type emerged as significant moderators, with numerical ability showing stronger correlations compared to general cognitive, verbal, and spatial abilities. Importantly, our study found that correlations in student samples were significantly higher than those in general samples. Our findings show a moderate positive association of SAI with IQ, unaffected by participant sex, publication year, administration order, neuroticism, and self-assessment method, yet significantly moderated by ability domain and sample type. Our results illustrate the importance of feedback in educational settings to help students accurately assess their cognitive abilities.

## 1. Introduction

Self-assessed intelligence (SAI) has been suggested to be related to important life outcomes, such as career decisions and life satisfaction (e.g., Neubauer and Hofer 2021; Zajenkowski et al. 2020), with incorrect estimates potentially leading to adverse outcomes, such as missed opportunities or failures in academic and professional contexts, due to the overestimation or underestimation of one’s capabilities (e.g., Ackerman and Wolman 2007). While one could argue that no-one knows their abilities better than the respective people themselves, previous evidence suggests otherwise, with correlations between SAI and psychometric intelligence ranging from *r* = −0.25 to *r* = 0.85 (Freund and Kasten 2012). Previous meta-analytic accounts revealed a moderate correlation between both variables (Freund and Kasten 2012; Mabe and West 1982; Zell and Krizan 2014). However, self-assessment accuracy was theorized to be influenced by various aspects related to characteristics of SAI measurement, participants, and test conditions (Freund and Kasten 2012; Zell and Krizan 2014).

For instance, previous evidence indicates that the validity of self-estimates (i.e., their correlation with objective tests) may be more accurate when specific (cognitive) abilities, as opposed to general ability, have to be self-estimated (Freund and Kasten 2012; Zell and Krizan 2014). In this vein, it has been speculated that lower accuracies of general cognitive ability estimates can be attributed to general abilities being more abstract and, therefore, harder to self-assess. Furthermore, some specific abilities, such as verbal, spatial, and numerical intelligence, have been suggested to be perceived as more salient in the general population (Furnham 2000), as opposed to other, possibly less-known, cognitive ability domains such as naturalistic intelligence. Therefore, it can be expected that correlations of self-estimates with cognitive ability may be differentiated according to the examined ability domain. For the purposes of this study, we opted for a broad definition of intelligence.

Importantly, in past research, the operationalization of intelligence between different studies has been observed to vary considerably (e.g., Freund and Kasten 2012). On the one hand, in terms of measuring intelligence, standard psychometric test instruments were typically used that have been developed within the framework of widely accepted intelligence theories, such as the CHC model (Cattell–Horn–Carroll model; Schneider and McGrew 2018) or its predecessor models. On the other hand, self-assessed intelligence was, in several cases, operationalized according to contested ideas of intelligence concepts, such as Gardner’s theory of multiple intelligences (Gardner 1983). This means in the context of the present paper that we mean results of standardized intelligence tests when referring to measured cognitive abilities (henceforth: IQ) but refer to a rather broad conceptualization of intelligence when it comes to SAI, which includes domains such as “naturalistic intelligence”, for which no standardized measures have been developed to date.

Previous evidence also shows that self-assessment accuracy improves when individuals evaluate their abilities by comparing themselves with others (Freund and Kasten 2012). Such improvements have been observed in studies where either relative labels (e.g., ”below average” and ”above average”, as seen in Hofer et al. 2022) were used or when participants were required to compare their abilities with those of a specific reference group (e.g., providing undergraduate students with their peer group’s average scores, thus facilitating direct comparisons, as seen in Furnham 2018). This observation may be rooted in interpersonal comparisons, providing a tangible frame of reference that may enhance precision in self-estimates (Festinger 1954). Therefore, it may be expected that relative response scales may facilitate more accurate self-assessments and, consequently, larger self-assessed intelligence and IQ correlations compared to absolute or mixed scales.

The well-documented over-reliance on student samples in the psychological literature (Thalmayer et al. 2020), in combination with the non-representativeness of such samples for the general population (e.g., Hanel and Vione 2016), indicates a necessity to examine the effects of sample type (i.e., whether a general population or student sample was used) on observed effect sizes. This is particularly important because psychology students may arguably have more substantial knowledge of both their general and specific abilities (e.g., due to mandatory or voluntary exposure to test instruments in the context of their studies). Thus, on the one hand, students’ prior experience with general and specific ability tests may lead to more accurate ability self-assessments, resulting in larger effects in student samples. On the other hand, range restriction in psychology students may be expected to lead to smaller student sample effects than in the general population.

Higher self-estimates of men compared to women in stereotypically “male” domains such as logical and spatial intelligence (e.g., Rammstedt and Rammsayer 2002; Stieger et al. 2010; Syzmanowicz and Furnham 2011) have led some researchers to speculate about the existence of a so-called Hubris–Humility Effect (e.g., Kaufman 2012; Storek and Furnham 2012). This term describes that male participants are more likely to overestimate (i.e., showing hubris) and female participants to underestimate (i.e., showing humility) their (specific) cognitive abilities. However, previous findings have been ambiguous, reporting that, even though women show a pattern of underestimation with a difference of over six IQ points in SAI and IQ scores (Reilly et al. 2022), this might not impact the SAI and IQ correlation, because correlation coefficients are independent of (systematic) misestimations.

Furthermore, personality differences have been hypothesized to cause sex differences in self-assessed intelligence. On the one hand, previous meta-analytical accounts (Howard and Cogswell 2018) showed that neuroticism is negatively associated with SAI, suggesting that individuals with higher neuroticism scores tend to estimate their intelligence lower. Moreover, larger self-estimates of men, compared to women, regarding spatial and logical abilities can (partly) be explained in terms of men’s lower neuroticism scores (Stieger et al. 2010). Specifically, individuals with lower neuroticism tend to perceive events and personal abilities more positively, which may plausibly generalize to their view of their own intelligence. However, previous findings have, in fact, been ambiguous, showing that neuroticism is negatively associated with self-estimates of fluid intelligence but may be unlikely to significantly impact the accuracy of these self-estimates when compared to objective test scores (Jacobs et al. 2012). Potential effects of neuroticism may be particularly interesting because it has been demonstrated to represent a suppressor of the association of SAI with other variables, such as trait-anger, in some studies (Zajenkowski and Gignac 2018). However, the association of neuroticism with the SAI–IQ link remains complex and needs further investigation.

On the other hand, it has previously been argued that SAI could be seen as a domain-specific form of self-efficacy (Howard and Cogswell 2018). Intuitively, one might expect a strong association between trait self-efficacy beliefs and SAI. Some studies have reported significant correlations between self-estimated abilities and self-efficacy beliefs (e.g., Brady-Amoon and Fuertes 2011), while others have found no such relationship (Ng and Earl 2008). Therefore, further investigation into the influence of self-efficacy beliefs on the relationship between SAI and psychometric intelligence is necessary.

Finally, it has been theorized that the administration order of self- vs. psychometrically assessed intelligence may play a role in the strength of the IQ and SAI correlation (Freund and Kasten 2012; Zell and Krizan 2014). This idea is consistent with reports of more modest and accurate self-assessments when psychometric IQ was assessed before compared to when it was assessed after SAI (Furnham 2018). Arguably, having just worked on an IQ test may anchor participant self-perceptions in reference to a more objective criterion. However, so far, the available empirical support for this idea is limited.

While many of these potentially moderating variables have previously been tested (e.g., Freund and Kasten 2012; Mabe and West 1982; Zell and Krizan 2014), their interactions with different analytical approaches in primary or meta-analytical studies have so far not been systematically examined. Study outcomes may vary considerably depending on which variables are analyzed and how these analyses are carried out (Voracek et al. 2019). For instance, choices about whether to include general or specific cognitive abilities and how to correct for measurement errors can impact results (Hunter and Schmidt 2004; Hedges and Olkin 1996). This multiverse of decisions can be examined using specification curve analyses (Simonsohn et al. 2019) and combinatorial meta-analyses (Olkin et al. 2012). Additionally, dissemination biases have been demonstrated to often lead to the inflated effects reported in the literature and declining strengths of effect sizes over time (Pietschnig et al. 2019), necessitating the use of multiple bias detection methods (Siegel et al. 2022; Carter et al. 2019). However, the generality of the SAI and IQ link in reference to the multiverse of scientific decision-making and its stability in terms of the decline effect and bias remains unclear to date.

Therefore, here, we aim to investigate the strength of the SAI and IQ association in healthy adults by (i) assessing the effects of potential moderating variables, (ii) establishing the generality vs. specificity of the effect according to which data are analyzed, and how these analyses are performed by using multiverse analyses, and (iii) examining possibly confounding dissemination biases.

## 2. Materials and Methods

The present meta-analysis was preregistered at the Open Science Framework at https://osf.io/xa2gp (accessed on 25 August 2024). Any deviations from preregistrations are documented at https://osf.io/kdsrx (accessed on 25 August 2024). The present study conforms to the PRISMA reporting guidelines (Moher et al. 2009), with the PRISMA checklist available at https://osf.io/mxa2f (accessed on 25 August 2024). Despite having been preregistered, analyses pertaining to self-efficacy were not performed in our study due to data unavailability.

### 2.1. Literature Search

First, we obtained the effect sizes of studies conducted from 1915 to 2010 from a previous meta-analysis (Freund and Kasten 2012). Second, we updated these data starting from 2011 using the following search string in the online databases PsychINFO, PSYNDEX, Scopus, and ISI Web of Science, Google Scholar, and Open Access Dissertation and Theses (oatd.org; from the latter two databases, only the first 100 hits were screened) to obtain potentially relevant studies: (cognitive ability OR intelligenc*) AND (estimate* OR perceive* OR self-apprais* OR self-assess* OR self-estimate* OR self-evaluat* OR self-perceive* OR self-rate*). We removed hits from ISI Web of Knowledge from the fields of computer science, artificial intelligence, engineering, electronics, robotics, physics, and mathematics. Third, we performed a cited reference search in ISI Web of Science for a previous meta-analysis on the SAI and IQ association (Freund and Kasten 2012). Fourth, we screened the reference lists of included studies to identify further potentially eligible studies.

Out of 25,683 hits, 19,218 titles and abstracts were screened following deduplication. Finally, 956 full-text articles were assessed to be appraised for potential inclusion. The original search was carried out in April 2020 and updated in July 2022 and May 2024. All articles retrieved through database searches (after excluding duplicates) and the reasons for exclusion during full-text screening are documented at https://osf.io/2rnuj (accessed on 25 August 2024).

### 2.2. Inclusion Criteria

Studies had to meet five inclusion criteria to be eligible for inclusion. First, studies had to report associations between SAI and IQ. Second, intelligence had to be assessed using a standardized intelligence test. Third, a direct measure of self-assessed cognitive ability had to be used (i.e., studies with indirect measures, such as self-assessed cognitive impairment, were excluded). Fourth, except for those studies that were included by Freund and Kasten (2012), all included studies must have been conducted after 2011. Fifth, participants had to be healthy adults (i.e., mean age > 18 years).

The first author [SP] screened titles, abstracts, and full texts for eligibility and coded the relevant information (see below). Ambiguities were resolved by discussion with an independent coder [JP].

### 2.3. Data Extraction

All studies were coded by the first author [SP]; coding included:

- Correlation coefficients: The relationship between SAI and IQ.
- Total sample sizes: The number of participants included in each study.
- Participant sex, categorized as (i) women only, (ii) men only, or (iii) mixed (both, women and men) sample.
- Sample type, categorized as (i) student sample (i.e., participants were university students), (ii) general population sample (participants were from the general public), or (iii) unknown sample type (i.e., the type of participants was not specified).
- Publication year: The year the study was published.
- Self-assessment type: This variable describes how participants rated their abilities and includes: (i) absolute scale (i.e., participants rated their abilities on a scale without comparing themselves to others, e.g., rating their ability on a scale 1 to 10 or with absolute terms, such as bad or good), (ii) relative scale without specific reference group (i.e., participants rated their abilities using relative labels, e.g., above average, below average), (iii) relative scale with specific reference group (i.e., participants rated their abilities using relative labels and in comparison to a specific group; e.g., participants are presented with the intelligence distribution of their peers and have to estimate their own cognitive abilities), or (iv) mixed scale (i.e., a combination of absolute and relative assessments, e.g., participants estimate their abilities on a scale with labels bad, average and good).
- Ability domain: The specific domain of ability being assessed, including (i) general: (i.e., overall cognitive ability), (ii) numerical (i.e., abilities related to math and number manipulation, e.g., number series, numerical reasoning, or arithmetic tasks), (iii) spatial (i.e., abilities related to spatial orientation and visualization, e.g., spatial analogies, spatial orientation, or paper folding tasks). (iv) verbal (i.e., abilities related to language and communication, e.g., word fluency, vocabulary knowledge, or verbal comprehension tasks) or (v) other cognitive ability (i.e., any other cognitive domain not covered by the above categories)
- Administration order: The order in which assessments were administered: (i) SAI first (i.e., self-assessment of ability was conducted before the IQ test), (ii) IQ first (i.e., the IQ test was conducted before the self-assessment of ability), or (iii) unknown (i.e., the order of administration was not specified.

Sample neuroticism mean (i.e., mean neuroticism values within sample).

- Self-efficacy mean (i.e., mean neuroticism values within sample).

We recorded intelligence tests and self-assessment reliabilities wherever available to adjust for effect unreliabilities in terms of a Hunter and Schmidt-type meta-analytical approach. When no reliabilities were reported, we (i) estimated values by averaging the given test reliabilities in the dataset or (ii) used the R package psychmeta (Dahlke and Wiernik 2019) to impute missing reliability values based on a bootstrap approach. In all, 178 self-assessment measure reliabilities and 131 intelligence test reliabilities had to be imputed.

In all, 10% of the presently coded studies were randomly selected and independently coded by another independent researcher. Interrater reliability yielded K = 0.96 (median: K = 1.00; values ranging from 0.47 to 1.00 between studies), indicating excellent agreement.

### 2.4. Data Analysis

Given that multiple studies reported more than one effect size of identical samples, we adopted a three-level meta-analytical approach to account for these dependencies. By using hierarchical linear modeling (HLM; Raudenbush and Bryk 2002), we preserved the entirety of the information, thus ensuring maximum statistical power (Assink and Wibbelink 2016).

In three-level meta-analytic models, three variance sources are examined, namely (i) sampling variance at level 1 (i.e., variability in effect sizes that arises from the fact that each study’s results are based on a sample of participants rather than the entire population), (ii) within-study, between-effect size variance at level 2 (i.e., variability in effect sizes within a single study, which accounts for differences in the effect sizes that are reported within the same study), and (iii) between-study variance at level 3 (i.e., variability in effect sizes between different studies, which captures the differences that arise from variations in study characteristics). This allows effect sizes to vary between participants, effect sizes, and studies (Assink and Wibbelink 2016). We used random-effects models to calculate summary effects (i.e., REML; Viechtbauer 2010).

Leave-one-out analyses, multiverse analyses, and most dissemination bias detection techniques are inappropriate for handling dependent data or three-level meta-analytic models. Consequently, we only used data of independent effect sizes in two-level models in these calculations. To this end, we averaged within-study correlations for applying dissemination bias techniques, along with leave-one-out analyses. In all, 115 independent data points were available for these examinations.

For our multiverse analyses, we calculated separate meta-analyses for each specification (see below). Thus, dependent effect sizes could be included as independent effect sizes if they did not co-occur in identical specifications. Therefore, only dependent effects with exact specifications were averaged for this approach. We used the resulting 200 effect sizes with distinct specifications for our specification curve and combinatorial meta-analyses.

To examine potential moderator effects, we extended the three-level model by adding regression terms to our model in a mixed-effects approach (Assink and Wibbelink 2016). Influences of participants’ sex, sample type, self-assessment type, cognitive ability type, administration order, and mean neuroticism were expected to take place on the effect size level (level 2), whilst publication year was hypothesized to explain variance at the study level (level 3). However, analyzing multiple potential moderators simultaneously risks introducing multicollinearity, preventing meaningful interpretation of results.

Therefore, following current recommendations (Assink and Wibbelink 2016; Hox 2010), we first assessed the effects of each potential moderator individually. This initial step involves separately evaluating the influence of each moderator in univariate models to determine the independent effect of each variable without accounting for the confounding influences of others. For categorical moderators—participant sex, sample type, self-assessment type, cognitive ability type, and administration order—we performed separate subgroup analyses using mixed-effects models. In these analyses, effect sizes were assigned to respective subgroups, and their means were compared using three-level random-effects models for within-subgroup estimates and fixed-effect models for between-subgroup comparisons. We conducted single linear precision-weighted meta-regressions for continuous moderators, specifically neuroticism and publication year.

Subsequently, we calculated a multiple regression using only those predictors that had shown significant influences in our bivariate analyses.

We used leave-one-out analyses to assess the stability of summary effect calculations and to identify potential leverage points. In these analyses, summary effects are calculated by removing a single effect size in each respective turn. In this vein, substantial numerical changes in summary effect estimations may indicate summary effect-distorting influences of respective effect sizes.

#### 2.4.1. Dissemination Bias

Dissemination biases have often been demonstrated to lead to inflated effects reported in the literature and declining strengths of effect sizes over time (Pietschnig et al. 2019). These time trends have been linked to selective reporting and dissemination bias, thus necessitating suitable detection methods to identify effects of (non-genuine) cross-temporal and bias-related effect changes (Siegel et al. 2022). Because there is no single bias detection method that clearly outperforms other available methods (Carter et al. 2019), this makes it necessary that (i) a reasonable number of detection methods that are based on different approaches is used (Siegel et al. 2022) and (ii) cross-temporal effect changes are investigated, to detect potential influences of confounding bias (Pietschnig et al. 2019).

Therefore, we presently used nine different methods to assess dissemination bias. Applying a large number of methods was deemed appropriate because different types of detection methods have been shown to possess differing sensitivities according to bias types (for detailed descriptions of bias detection methods, calculations, and inference criteria, see Siegel et al. 2022). Only effect sizes from published studies were used in these analyses. We used five methods to assess small-study effects: (i) Contour-enhanced funnel plot inspection (Peters et al. 2008; Kossmeier et al. 2020), (ii) Sterne and Egger’s (2005) regression approach, (iii) Duval and Tweedie’s trim-and-fill method (Duval and Tweedie 2000a, 2000b), (iv) Begg and Mazumdar’s (1994) rank correlation, and (v) PET–PEESE (Stanley and Doucouliagos 2014).

Three *p*-value-based methods, that are particularly useful to detect effects of *p*-hacking and allow *p*-value-based summary effect estimation, were used: (i) *p*-curve (Simonsohn et al. 2014a, 2014b), (ii) *p*-uniform (van Assen et al. 2015), (iii) as well as *p*-uniform* (van Aert and van Assen 2018).

Finally, we used the test for excess significance according to the approach of Ioannidis and Trikalinos (2007).

Detailed descriptions of these methods are provided in the appendix on the OSF. Following standard analytic approaches, *p*-values < .10 were assumed to be indicative of bias in publication bias analyses (see Siegel et al. 2022).

#### 2.4.2. Multiverse Analysis

To assess the generality of our findings, we used specification curve and combinatorial meta-analyses in our multiverse approach to assess the influence of various analytical choices in terms of which data are analyzed and how this is done. While many potentially moderating variables have previously been tested (e.g., Freund and Kasten 2012; Mabe and West 1982), their interactions with different analytical approaches in primary or meta-analytical studies have so far not been systematically examined. There are many (reasonable) ways in which primary or meta-analytical studies can be designed. Study outcomes may vary considerably depending on which variables are analyzed and how these analyses are performed (Voracek et al. 2019).

For example, when meta-analytically examining SAI and IQ associations, for some researchers it may seem reasonable only to include data from participants who were asked to estimate their general cognitive ability, whilst others would argue that estimates of specific abilities should be included as well (i.e., representing two different and equally reasonable ways in terms of which data to analyze). Presently, we used five “which” factors, comprising participants’ sex, sample type, self-assessment type, ability domain, and administration order.

When it comes to the question of how to analyze the data, some researchers might argue that observed correlations between SAI and IQ should be corrected to account for measurement error or unreliability (i.e., following the meta-analytical approach of Hunter and Schmidt 2004) to correct for effect underestimation. Others might argue that the Hunter and Schmidt approach leads to inflated effect estimates and various other confounders (e.g., Wiernik and Dahlke 2020) and may, therefore, prefer Hedges and Olkin’s analytical approach (Hedges and Olkin 1996) to avoid effect inflation. We used two “how factors”, comprising effect size metric and synthesis type. Out of the resulting 1920 “which” (4 × 4 × 5 × 6 × 4) by 15 (3 × 5) “how” factor combinations (see Table 1), totaling 28,800 reasonable specifications, 4980 specifications yielded at least two independent effect sizes, thus allowing summary effect calculations.

In addition to the descriptive specification curve, we used a bootstrapping method to assess the significance of our observed specification curve. This was carried out by drawing 1000 random samples, assuming that the null hypothesis was true and study features were fixed, thus resulting in a nil-effect specification curve of point estimates with an associated 95% confidence interval. Comparing the observed with the nil-effect specification curve allowed us an inferential assessment of the observed effect distribution.

Arguably, specification curve analyses may be insufficient to provide evidence about potential moderators because not all moderators may have been conceptually identified before analyses. We used combinatorial meta-analyses (Olkin et al. 2012) to alleviate this concern by examining all possible instead of only reasonable study combinations. According to this idea, patterns in summary effect estimations as a function of summary effect strength and between-studies heterogeneity make it possible to detect influences of moderating variables that had not been considered before analysis.

In this vein, 2^k^ − 1 possible combinations should be examined (Olkin et al. 2012). However, the resulting large number of necessary summary effect estimations in most meta-analyses (presently 2200 − 1) typically exceeds the computational power of standard computer hardware. Consequently, we presently selected 100,000 data subsets at random to calculate our combinatorial analyses. In the resulting GOSH (graphical display of study heterogeneity) plot, influential cases and potential subgroup effects can be identified due to unobserved between-studies heterogeneity. Here, we used a stratified approach that oversamples studies with the smallest and largest effects to have a maximum probability of assessing outlier effects (see Voracek et al. 2019).

We used the open-source software R version 4.3.1 for data analyses. Our analytical R code is available from https://osf.io/9hp38 (accessed on 25 August 2024), and all packages used are listed at https://osf.io/xq3zk (accessed on 25 August 2024). Dissemination bias methods were calculated using the online application MetaShine (https://the-meta-analysis-project.shinyapps.io/MetaShine (accessed on 25 August 2024); (Siegel et al. 2021), excepting PET–PEESE, contour-enhanced funnel plots and *p*-curve analyses). For all multiverse analyses, the analytical R Code is available at https://osf.io/nkv46 (accessed on 25 August 2024).

## 3. Results

### 3.1. Final Sample

**Figure 1:**
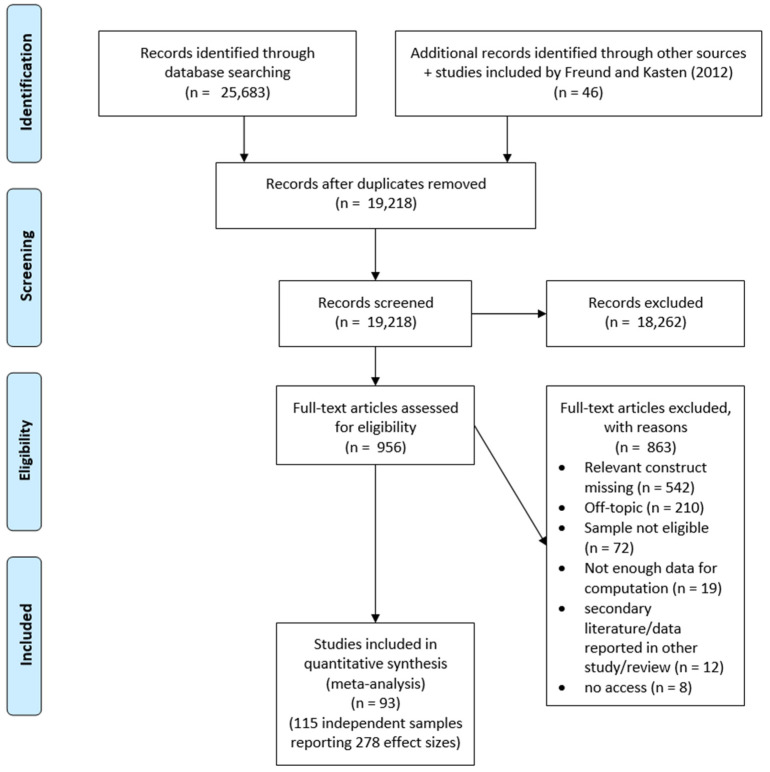
Flowchart of study inclusion.

In all, 278 effect sizes from 115 independent samples reported in 93 studies (*N* = 36,833; *n*-range = 13 to 13,690) were included in our meta-analysis. Notably, 94% of these effect sizes were positive, with correlations ranging from *r* = −0.25 to *r* = 0.70. A flowchart of study inclusion is provided in Figure 1. Correlation coefficients were predominantly reported for mixed-sex samples (246 effect sizes), with only 16 effect sizes representing female-only and another 16 male-only samples. Effect sizes were based on samples from the United Kingdom (61), the United States (57), Germany (33), Poland (22), Austria (20), Australia (16), Canada (15), Italy (9), Greece (7), Switzerland (6), Turkey (6), the Netherlands (4), Russia (4), Sweden (2), and the remaining 16 effect sizes were from Belgium, China, France, Norway, multiple countries, or an unspecified country. Most studies (i.e., 193 effect sizes) employed single-item measures for SAI, while a minority utilized multi-item scales ranging from 2 to 35 items. The full dataset is available at https://osf.io/usj8b (accessed on 25 August 2024). Results from all pair-wise comparisons from moderator analyses are numerically detailed at https://osf.io/fqrvm (accessed on 25 August 2024).

### 3.2. Three-Level Meta-Analysis

We observed a moderate positive correlation between self-assessed intelligence and IQ (*r* = 0.297; 95% CI [0.271, 0.323]; t(277) = 21.160, *p* < .001). Substantial between-studies heterogeneity (*Q*(277) = 1531.474, *p* < .001; I^2^ = 84.69%) suggested the presence of moderating factors. Variance components were differentiated in terms of level, with 15.31% originating from the participant level, 46.19% from the effect size level, and 38.80% from the study level. A comparison between the full three-level meta-analytic model and a reduced one, excluding levels 2 and 3, demonstrated a superior fit of the former (top of Table 2).

### 3.3. Moderator Analysis

We provide descriptive statistics of summary effects of all subgroup analyses in Table 3.

#### 3.3.1. Participant Sex

We ran subgroup analyses using mixed-effects models to investigate differences in correlations between SAI and IQ depending on whether correlations were reported for a women-only, men-only, or mixed sample. We observed no significant influences of participant sex (*Q*(2) = 1.425, *p* = .490) on the SAI and IQ link.

#### 3.3.2. Sample Type

SAI and IQ associations differed significantly depending on whether correlations were reported for a student, general, or unknown sample (*Q*(2) = 11.818, *p* = .003). Pairwise comparisons showed that correlations in student samples were stronger than those reported in general samples (*Q*(1) = 11.601, *p* < .001).

#### 3.3.3. Self-Assessment Type

SAI and IQ associations differed significantly from each other according to the self-assessment method (*Q*(4) = 30.106, *p * < .001). Pairwise comparisons showed that correlations were strongest when either type of relative scale was employed compared to an absolute scale (absolute scale vs. reference group: *Q*(1) = 6.958, *p* = .008; absolute scale vs. relative scale: *Q*(1) = 9.026, *p* = .003). Furthermore, employing a relative scale resulted in stronger correlations than a mixed scale (*Q*(1) = 3.981, *p* = .046).

#### 3.3.4. Ability Domain

The correlation between SAI and IQ was significantly influenced by the scrutinized ability domain (*Q*(4) = 29.531, *p * < .001). Pairwise comparisons showed that correlations were strongest when numerical ability was assessed, significantly exceeding correlations in all other domains (numerical vs. general: *Q*(1) = 22.677, *p* < .001; numerical vs. spatial: *Q*(1) = 25.223, *p* < .001; numerical vs. verbal: *Q*(1) = 13.404, *p* < .001; numerical vs. other: *Q*(1) = 26.246, *p* < .001). Additionally, general cognitive ability showed stronger correlations than other cognitive abilities (general vs. other: *Q*(1) = 4.376, *p* = .036).

#### 3.3.5. Administration Order

The correlation between SAI and IQ differed significantly according to the administration order (*Q*(2) = 7.973, *p* = .019). Pairwise comparisons showed that associations were significantly lower when the administration order was unknown compared to when the administration order was specified (unknown vs. test-first: *Q*(1) = 7.209, *p* = .007, unknown vs. estimate-first: *Q*(1) = 5.297, *p* = .022).

#### 3.3.6. Publication Year and Neuroticism

To examine the potential impact of publication year and neuroticism, we conducted single linear precision-weighted meta-regressions. However, our analyses did not reveal any significant effects of either publication year (*F*(1, 276) = 3.008, *p* = .084, β = −0.002) or neuroticism (*F*(1, 50) = 0.247, *p* = .622, β = 0.107) on the correlation between SAI and IQ.

#### 3.3.7. Multiple Regression Analysis

We entered dummy coded sample type, self-assessment type, ability domain, and administration order as predictors in our multiple regression model (Table 4). The sample type and ability domain significantly influenced effect sizes, whilst there were no effects of self-assessment type or administration order. Specifically, correlations were significantly weaker for general samples compared to student samples (β = −0.081, *p* = .003). Additionally, numerical ability showed significantly larger associations compared to general cognitive ability (β = 0.178, *p * < .001), whilst other cognitive abilities showed significantly weaker ones (β = −0.069, *p* = .023).

Between-studies heterogeneity was lower than in our model without moderators but remained substantial, yielding *I*² = 80.04% (*Q*(266) = 1176.401, *p * < .001). This indicates that there may be further potentially meaningful moderators that have not been accounted for in these analyses (Higgins et al. 2003). Variances were attributable to participants (19.96%), effect size (41.39%), and study level (38.65%). Again, model fit comparisons favored the full model over the reduced versions (bottom of Table 2).

### 3.4. Sensitivity Analysis

There were no substantial changes in summary effects when individual effect sizes were omitted from analyses.

### 3.5. Dissemination Bias

**Figure 2:**
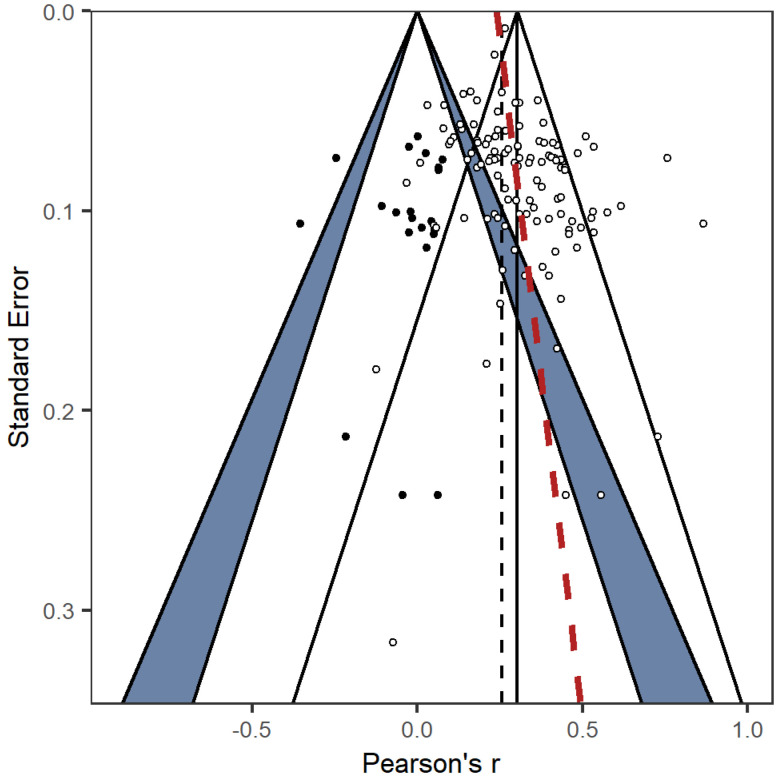
Contour-enhanced funnel plot based on a two-level model. The vertical solid line represents the observed effect; the dashed black line represents an adjusted summary effect according to trim-and-fill; the red dashed line represents Egger’s regression line; effect sizes in the blue area of the zero-effect funnel (left-hand funnel) are significant at *p* < .05, effect sizes outside of zero-effect funnel are significant at *p* < .01; circles represent observed effect sizes, black dots represent imputed effect sizes according to the trim-and-fill method.

Visual examination of contour-enhanced funnel plots (Peters et al. 2008; Kossmeier et al. 2020) revealed some evidence for asymmetry (Figure 2). Consistent with this interpretation, both the regression approach by Sterne and Egger (2005); z = 2.850, *p* = .004; see regression line in Figure 2) and Begg and Mazumdar’s (1994) rank correlation test (Kendall’s τ = 0.188, *p* = .003) yielded significant results. Similarly, trim-and-fill (Duval and Tweedie 2000a, 2000b) indicated 21 missing studies on the left side of the funnel plot. The difference between adjusted and observed summary effects did not exceed 20%, though, thus yielding no bias evidence according to established benchmarks (Siegel et al. 2022). Results of PET–PEESE (Stanley and Doucouliagos 2014) were consistent with trim-and-fill results, showing no significant bias indication.

**Figure 3:**
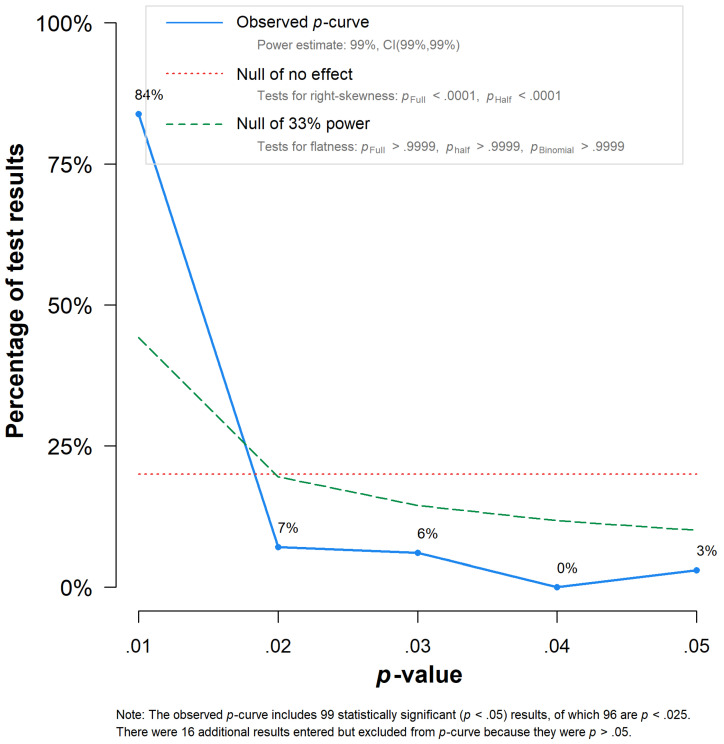
*p*-curve. The plot is based on a two-level model.

Our *p*-curve analyses (Simonsohn et al. 2014a, 2014b) revealed a significantly right-skewed conditional *p*-value distribution (*p * < .001), indicating no evidence for *p*-hacking-related bias as well as no indication of insufficient evidential value for the observed non-null effect (*p* > .999; Figure 3). Non-significant results of our *p*-uniform (van Assen et al. 2015) bias analysis were consistent with this result (*p* = .998). *p*-curve-, *p*-uniform-, and *p*-uniform*-based (van Aert and van Assen 2018) effect estimations showed summary effects of similar strength as our conventional three-level model-based estimation, yielding effects of *r* = 0.30, *r* = 0.31, and *r* = 0.27, respectively.

Finally, the test of excess significance did not show significant evidence for an overrepresentation of significant primary studies in the available literature (*p* = .567).

### 3.6. Multiverse Analyses

**Figure 4:**
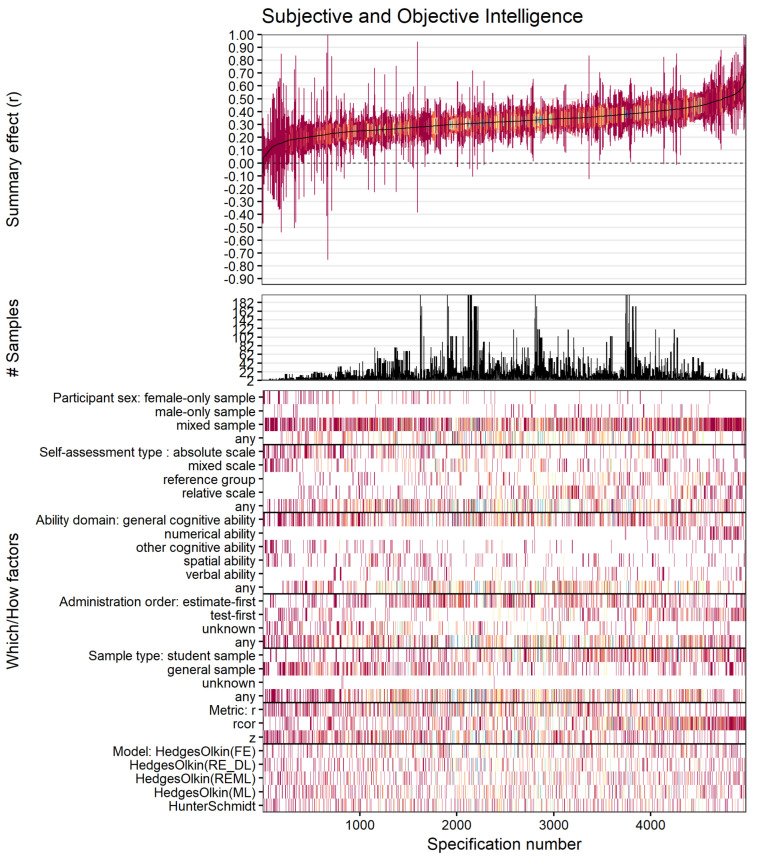
Descriptive meta-analytic specification-curve plot. The top panel of the plot represents the observed summary effects according to effect strengths and their corresponding 95% confidence intervals (interval overlap with the horizontal line indicating non-significant summary effects). The middle panel shows the associated number of samples within the specification (at least two effects, respectively). The plot’s bottom panel shows the combinations of “Which” and “How” factors. Warm colors (red, orange, yellow) indicate low precision, and cool colors (blue, green, purple) indicate high precision of effect estimates.

All but 185 summary effects from 4980 different specifications yielded positive significant summary effects (i.e., 96%; *r* range: −0.092 to 0.706). Summary effects averaged *r* = 0.321, with a median value of 0.321 (interquartile *r* range: 0.259 to 0.378). Consistent with our moderator analyses, specification patterns revealed larger effects for numerical ability correlations compared to the other abilities and student samples compared to general samples (Figure 4).

**Figure 5:**
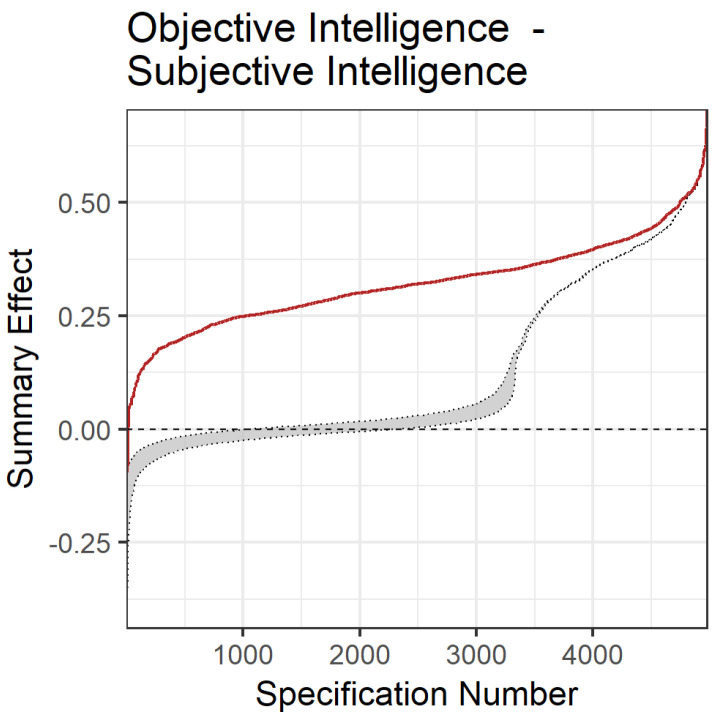
Inferential meta-analytic specification plot. Red = specification curve of observed meta-analytic summary effects; grey = bootstrap-based specification curve with confidence interval under the assumption of a nil-effect.

The observed specification curve differed substantially from the bootstrapped nil-solution in our inferential meta-analytic specification plot (Figure 5), thus supporting the meaningfulness of the observed association.

**Figure 6:**
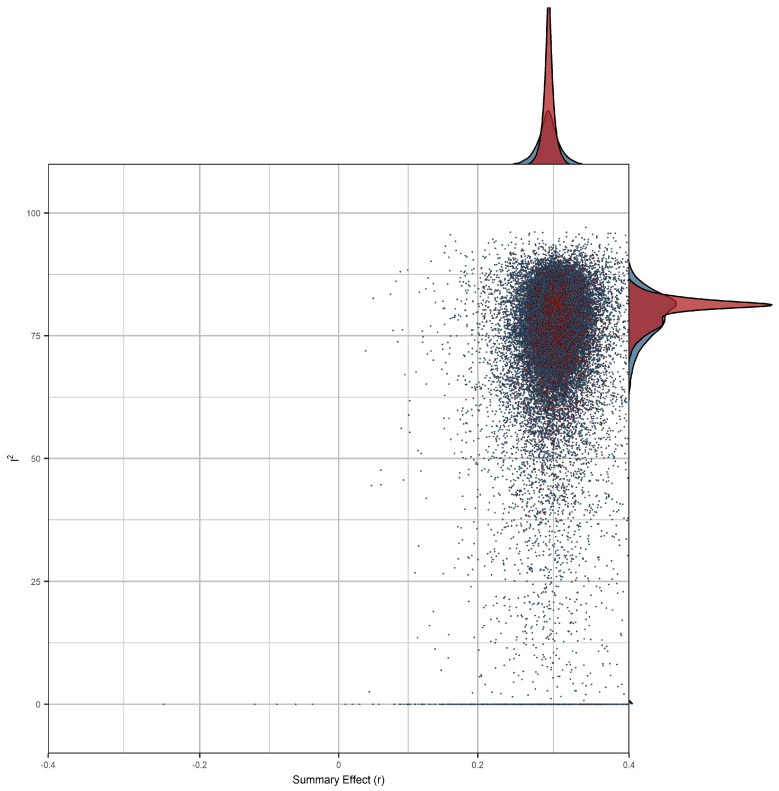
GOSH-plot for combinatorial meta-analysis. 100,000 random subsets from our combinatorial meta-analyses, where each dot represents the summary effect of a random subset of studies. To aid visual interpretation, estimates from subsets, including effect sizes from two studies that contributed disproportionally to between-studies heterogeneity, are highlighted in red (all other subsets are represented by blue dots). These points, while notable, did not lead to substantial changes in summary effects.

The results of combinatorial meta-analyses indicated substantial heterogeneity in most specifications (Figure 6). However, between-studies heterogeneity appeared to be unaffected by outliers. Moreover, summary effects averaged *r* = 0.306 (median: *r* = 0.307), and 50% of observed summary effects ranged from r = 0.300 to 0.313, thus broadly conforming to the specification curve and standard analysis results.

## 4. Discussion

Here, we provide evidence for a positive, moderate, and remarkably stable relationship between self-assessed and psychometrically assessed intelligence. This relationship generalized across participant sex, self-assessment type, and administration order. However, the association was differentiated according to ability domain and sample types. Furthermore, our multiverse analysis corroborated the remarkable generality of the observed positive SAI and IQ association. In all, 96% out of almost 5000 specifications indicated a significant positive link, with the majority of effects yielding a moderate effect strength, averaging *r* = 0.32. Although there was some evidence for effect inflation due to publication bias, the stability of the observed moderate effect does not appear to be substantially affected, even when accounting for these biases. Our results present several points of interest, as discussed below.

First, our analysis revealed the strongest SAI–IQ correlations for numerical abilities compared to all other investigated domains. General cognitive abilities yielded weaker associations compared to numerical abilities but were stronger than those of other specific abilities (e.g., spatial ability; Furnham 2000). It has previously been argued that the differentiation in terms of effect strength may be possibly due to individuals typically having had more opportunities in real-life contexts to obtain immediate feedback about their numerical task performance relative to their peers (e.g., when having to perform mental arithmetic in everyday tasks, such as splitting a bill three ways when dining out or when calculating a tip), compared to other cognitive abilities (e.g., Freund and Kasten 2012; Neubauer et al. 2018). Other products of cognitive abilities, such as spatial task performance, may receive less immediate feedback, thus making it more difficult for individuals to connect to real-life situations. This finding suggests that numerical tasks, often integrated into daily activities, allow for direct and frequent feedback, thus enhancing SAI and IQ correlations. Consequently, educational institutions may be advised to enhance feedback opportunities to ensure that students receive regular feedback on various cognitive tasks, not just numerical ones. This could involve giving feedback on a variety of tasks (e.g., reading and interpreting instructions) that reflect real-life scenarios beyond numerical abilities (e.g., spatial ability) and which can help students to obtain a better idea about their performance in certain cognitive abilities.

Second, our analysis revealed significant differences in the strength of correlations between different sample types. SAI and IQ correlations were significantly stronger in student samples compared to general population samples. This is perhaps our most surprising result, as a previous meta-analysis (Freund and Kasten 2012) did not find significant differences between student and general samples in SAI-IQ associations. However, this finding is consistent with the expectation that students, due to their prior experiences with psychometric measures and their substantial knowledge of their abilities, may be more accurate in self-assessing their IQ, resulting in stronger correlations between SAI and IQ. Because it is likely that feedback and prior experience with psychometric measures might enhance self-assessment accuracy, it seems plausible that providing not only diverse but also frequent and structured feedback might be beneficial in educational settings. By promoting self-awareness and helping students to become more aware of their cognitive strengths and weaknesses through structured self-assessment exercises, educators can aid them in making better-informed career decisions.

Third, there was no convincing evidence for influences of the use of different scale types on the SAI and IQ link. Strength differences in subgroup analyses disappeared in our specification curve analyses as well as when influences of other variables were accounted for in our multiple regression analyses. This observation contrasts with Festinger’s social comparison theory, which posits that people rely on social comparisons to enhance self-evaluation accuracy (Festinger 1954), which should presumably be triggered using relative scales. Our findings indicate that social comparisons may not be as effective as expected for self-evaluations of intelligence. Alternatively, mere modifications in scale wording may be insufficient to initiate social comparison processes effectively.

Fourth, subgroup analysis-based correlation strength differences due to scale administration order disappeared when ability domain, self-assessment type, and sample type were controlled for in a multiple regression analysis. This contrasts with findings from prior studies (Freund and Kasten 2012; Furnham 2018). Our results suggest that test administration order might not fundamentally alter long-standing self-perceptions of intelligence, thus conforming with previous findings indicating that SAI remains relatively stable over time (Cruise and Lewis 2006; Freund and Kasten 2012; Zell and Krizan 2014).

Fifth, mean sample neuroticism did not significantly moderate the relationship between SAI and IQ. This is in line with previous findings, suggesting that, even though neuroticism itself is associated with self-assessed fluid intelligence, it does not affect SAI’s relationship with fluid intelligence (Jacobs et al. 2012). Therefore, neuroticism does not appear to impact the association between SAI and IQ.

Sixth, our analyses did not reveal any significant influences of participant sex. Previous research indicated that women report lower self-estimates in certain types of cognitive abilities compared to men (Storek and Furnham 2012). However, systematic sex differences in self-estimates do not necessarily impact their correlation with another variable (i.e., IQ). This is in line with previous findings that have shown no sex differences in SAI–IQ correlations when solely looking at the correlation as a measure of accuracy (Reilly et al. 2022). However, a systematic pattern of underestimation in females was observed when examining the actual difference between SEI and IQ. Therefore, it is important to consider that sex differences may still be present in the accuracy of self-estimates.

Finally, we observed some evidence of dissemination bias in our data. Specifically, three out of nine used methods suggested influences of confounding bias, although influences of bias appeared to be modest at best. This demonstrates the stability of the SAI and IQ link, even when allowing for some influences of dissemination bias.

## 5. Limitations

Some limitations of our meta-analysis need to be acknowledged. First, our search strategy primarily focused on English databases, potentially introducing language bias. Although we included one German and one Russian study, we did not specifically search non-English databases. This may have led to an underrepresentation of relevant non-English-language studies.

Second, there was considerable heterogeneity between effect sizes even when moderators were accounted for. This indicates that further systematic influences of moderator variables remain to be revealed. However, evidence from our specification curve and combinatorial meta-analyses indicate stability of the observed effect according to different (reasonable) specifications.

Third, 226 out of 278 effect sizes were derived from studies conducted in WEIRD (Western, Educated, Industrialized, Rich, and Democratic) countries. This overrepresentation may limit the generalizability of our findings to non-WEIRD populations. Future research should aim to include more diverse samples to ensure broader applicability of the results.

Fourth, it cannot be ruled out that our observation of no meaningful influences of neuroticism on the SAI and IQ link may be due to restricted variability of neuroticism means within samples.

## 6. Conclusions

We provide here evidence for a moderate positive association of self-assessed intelligence with psychometric intelligence in healthy adults in the largest meta-analysis to date. The SAI and IQ link shows a remarkable generality across several moderators, analytical approaches, and their combinations but appears to be differentiated according to intelligence domain and sample type. This underscores the importance of frequent and broad feedback in educational settings to help students accurately assess their cognitive abilities, ultimately guiding better-informed career decisions.

## Figures and Tables

**Table 1:** Overview of “which” and “how” factors.

Variable	Variable Level
**“Which” factors**	
Participant sex	Women-only sample
	Men-only sample
	Mixed sample
	Any sample
Sample type	Student sample
	General sample
	Unknown sample type
	Any sample type
Method of self-assessment	Absolute scale
	Mixed scale
	Relative scale incl. specific reference group
	Relative scale
	Any method
Ability domain	General cognitive ability
	Numerical ability
	Spatial ability
	Verbal ability
	Other abilities
	Any ability domain
Administration order	Estimate-first
	Test-first
	Unknown
	Any order
**“How” factors**	
Effect size	Pearsons *r*
	Unreliability corrected *r*
	Fishers’ *z*
Estimator type	Hedges Olkin-typed approach: FE
	Hedges Olkin-typed approach: DL
	Hedges Olkin-typed approach: REML
	Hedges Olkin-typed approach: ML
	Hunter Schmidt-typed approach

**Table 2:** Model fit according to Likelihood-ratio-tests.

	*df*	LRT	AIC	BIC	*p*	*Q*
**Full and reduced three-level meta-analytic model**						
Full	3		−180.340	−169.468		1531.474
Without level two (within-study variance)	2	228.667	46.326	53.574	<.001	1531.474
Without level three (between-study variance)	2	44.684	−137.656	−130.408	<.001	1531.474
**Full and reduced three-level mixed-effects model (moderator model)**						
Full	14		−219.154	−168.985		1176.4014
Without level two (within-study variance)	13	126.403	−94.750	−48.165	<.001	1176.4014
Without level three (between-study variance)	13	36.568	−184.585	−138.000	<.001	1176.4014

**Table 3:** Results and descriptive statistics of subgroup analyses.

	Summary Effect (r)	SE	95% CI	*p*-Values	*Q*	I² (%)	Within-Study, τ^2^	Between Studies, σ^2^
Overall (*k* = 278)	0.297	0.015	[0.271, 0.323]	<.001	1531.474	84.69	0.012	0.014
Participant sex								
Women-only sample (*k* = 16)	0.264	0.051	[0.170, 0.355]	<.001	42.809	47.99	0.006	0.017
Men-only sample (*k* = 16)	0.344	0.055	[0.246, 0.435]	<.001	37.940	62.20	0.026	0.000
Mixed sample (*k* = 246)	0.294	0.015	[0.266, 0.321]	<.001	1446.278	86.03	0.015	0.012
Sample type								
Student sample (*k* = 162)	0. 332	0.017	[0.303, 0.361]	<.001	657.824	75.76	0.014	0.007
General sample (*k* = 113)	0.246	0.024	[0.198, 0.293]	<.001	779.085	88.99	0.015	0.013
Unknown sample type (*k* = 3)	0.242	0.142	[−0.031, 0.481]	.082	13.667	89.55	0.001	0.036
Method of self-assessment								
Absolute scale (*k* = 71)	0.246	0.029	[0.191, 0.299]	<.001	433.441	90.33	0.008	0.020
Mixed scale (*k* = 73)	0.281	0.027	[0.232, 0.330]	<.001	449.855	83.95	0.017	0.010
Relative scale incl. specific reference group (*k* = 66)	0.350	0.032	[0.294, 0.404]	<.001	299.593	78.20	0.016	0.011
Relative scale (*k* = 68)	0.341	0.019	[0.308, 0.372]	<.001	215.649	68.46	0.015	0.000
Ability domain								
General cognitive ability (*k* = 132)	0.305	0.016	[0.276, 0.334]	<.001	505.790	73.58	0.004	0.014
Numerical ability (*k* = 24)	0.434	0.027	[0.390, 0.476]	<.001	66.841	65.73	0.011	0.011
Spatial ability (*k* = 37)	0.246	0.033	[0.185, 0.306]	<.001	234.631	82.12	0.010	0.013
Verbal ability (*k* = 33)	0.310	0.029	[0.257, 0.360]	<.001	167.322	81.58	0.016	0.004
Other abilities (*k* = 52)	0.227	0.037	[0.158, 0.295]	<.001	341.451	88.88	0.010	0.019
Administration order								
Estimate-first (*k* = 108)	0.307	0.016	[0.279, 0.335]	<.001	373.025	71.92	0.012	0.002
Test-first (*k* = 87)	0.338	0.029	[0.287, 0.386]	<.001	566.692	83.83	0.011	0.025
Unknown (*k* = 83)	0.241	0.027	[0.191, 0.290]	<.001	521.484	87.66	0.009	0.016

Results from fitting a three-level meta-analytic model, consisting only of an intercept representing the overall effect of each subgroup, testing the null hypothesis of a zero effect. Model parameters were estimated using the REML method. *Q*: Cochran’s *Q* test statistic for heterogeneity, I^2^: proportion of observed variance due to true heterogeneity.

**Table 4:** Results of three-level mixed-effects multiple regression.

Variable	*k*	*b*	*SE*	95% *CI*	*p*
Intercept		0.308	0.033	[0.248, 0.365]	<.001
Sample type ^1^					
General sample	113	−0.081	0.027	[−0.134, −0.028]	.003
Unknown sample	3	0.000	0.103	[−0.201, 0.200]	.998
Self-assessment type ^2^					
Relative scale	68	0.055	0.035	[−0.014, 0.123]	.119
Reference group	66	0.059	0.038	[−0.016, 0.133]	.125
Mixed scale	73	0.016	0.034	[−0.052, 0.083]	.646
Ability domain ^3^					
Numerical ability	24	0.178	0.034	[0.113, 0.242]	<.001
Spatial ability	37	−0.038	0.032	[−0.101, 0.025]	.237
Verbal ability	33	0.019	0.031	[−0.042, 0.080]	.541
Other abilities	52	−0.069	0.030	[−0.127, −0.010]	.023
Administration order ^4^					
Test-first	87	0.220	0.030	[−0.038, 0.081]	.476
Unknown	83	−0.050	0.032	[−0.112, 0.012]	.114
**Variance component**					
Within-study, τ^2^	0.009				
Between studies, σ^2^	0.010				

^1^ Reference category: Student sample (*k* = 162). ^2^ Reference category: Absolute scale (*k* = 71). ^3^ Reference category: General cognitive ability (*k* = 132). ^4^ Reference category: Estimate-first (*k* = 108).

## Data Availability

The original data presented in the study are openly available at https://osf.io/usj8b (accessed on 25 August 2024).
